# Predicting acute kidney injury risk in acute myocardial infarction patients: An artificial intelligence model using medical information mart for intensive care databases

**DOI:** 10.3389/fcvm.2022.964894

**Published:** 2022-09-07

**Authors:** Dabei Cai, Tingting Xiao, Ailin Zou, Lipeng Mao, Boyu Chi, Yu Wang, Qingjie Wang, Yuan Ji, Ling Sun

**Affiliations:** ^1^Department of Cardiology, The Affiliated Changzhou No. 2 People’s Hospital of Nanjing Medical University, Changzhou, China; ^2^Graduate School of Dalian Medical University, Dalian Medical University, Dalian, China

**Keywords:** acute myocardia infarction, acute kidney injury, machine learning, random forest, area under the receiver operating characteristic curve

## Abstract

**Background:**

Predictive models based on machine learning have been widely used in clinical practice. Patients with acute myocardial infarction (AMI) are prone to the risk of acute kidney injury (AKI), which results in a poor prognosis for the patient. The aim of this study was to develop a machine learning predictive model for the identification of AKI in AMI patients.

**Methods:**

Patients with AMI who had been registered in the Medical Information Mart for Intensive Care (MIMIC) III and IV database were enrolled. The primary outcome was the occurrence of AKI during hospitalization. We developed Random Forests (RF) model, Naive Bayes (NB) model, Support Vector Machine (SVM) model, eXtreme Gradient Boosting (xGBoost) model, Decision Trees (DT) model, and Logistic Regression (LR) models with AMI patients in MIMIC-IV database. The importance ranking of all variables was obtained by the SHapley Additive exPlanations (SHAP) method. AMI patients in MIMIC-III databases were used for model evaluation. The area under the receiver operating characteristic curve (AUC) was used to compare the performance of each model.

**Results:**

A total of 3,882 subjects with AMI were enrolled through screening of the MIMIC database, of which 1,098 patients (28.2%) developed AKI. We randomly assigned 70% of the patients in the MIMIC-IV data to the training cohort, which is used to develop models in the training cohort. The remaining 30% is allocated to the testing cohort. Meanwhile, MIMIC-III patient data performs the external validation function of the model. 3,882 patients and 37 predictors were included in the analysis for model construction. The top 5 predictors were serum creatinine, activated partial prothrombin time, blood glucose concentration, platelets, and atrial fibrillation, (SHAP values are 0.670, 0.444, 0.398, 0.389, and 0.381, respectively). In the testing cohort, using top 20 important features, the models of RF, NB, SVM, xGBoost, DT model, and LR obtained AUC of 0.733, 0.739, 0.687, 0.689, 0.663, and 0.677, respectively. Placing RF models of number of different variables on the external validation cohort yielded their AUC of 0.711, 0.754, 0.778, 0.781, and 0.777, respectively.

**Conclusion:**

Machine learning algorithms, particularly the random forest algorithm, have improved the accuracy of risk stratification for AKI in AMI patients and are applied to accurately identify the risk of AKI in AMI patients.

## Introduction

Ischemic heart disease is a significant contributor to mortality in the global population, which is one of the leading causes of disability-adjusted life years (DALYs) in middle-aged and elderly patients (1). Acute myocardial infarction (AMI) is the most serious type of ischemic heart disease, which is one of the causes of Acute kidney injury (AKI) in patients. AKI occurs in a certain proportion of hospitalized patients with AMI. Studies have shown that the incidence of AKI during hospitalization in AMI patients ranges from 7.1 to 29.3% (2–4). The occurrence of AKI during hospitalization was independently associated with increased in-hospital mortality and long-term mortality post AMI (5–13). Several studies have also shown that AKI is associated with a significant increase in in-hospital mortality. Because there is unexpected and life-threatening characteristic of AMI, early identification of risk factors for AKI in patients with AMI is critical to improving overall prognosis, which can benefit patient management and overall treatment planning (14).

Machine learning is an important supporting technology for artificial intelligence. Machine learning is an algorithm that allows computers to “learn” automatically, analyze and construct models from data, and then use the models to make predictions for new samples. Machine learning predictive models are useful tools for identifying potential risk factors and predicting the occurrence of adverse events (15). In recent years, machine learning algorithms have been used increasingly in cardiovascular diseases. Combination with clinical big data, machine learning could help doctors predict risk accurately, therefore choose personalized medical treatment for patients. Than et al. developed a machine learning model and it could provide an individualized and objective assessment of the likelihood of myocardial infarction (16). Khera et al. reported three machine learning models which was developed with patients from the American College of Cardiology Chest Pain-MI Registry. They found that XGBoost and meta-classifier models offered improved prediction performance for high-risk individuals (17). Advanced machine learning methods were also used to predict the risk of tachyarrhythmia after AMI. The artificial neural network (ANN) model reached the highest accuracy rate, which is better than traditional risk scores (18). These studies indicated that machine learning is a reliable novel method for the clinic. Therefore, they broaden the new horizons for clinical researches.

MIMIC is a large, single-center, open-access database. MIMIC-III includes data on more than 58,000 admissions to Beth Israel Deaconess Medical Center in Boston from 2001 to 2012, including 38,645 adults and 7,875 newborns (19, 20). MIMIC-IV includes data from 524,740 admissions of 382,278 patients at the center from 2008 to 2019 (21, 22). The clinical records include demographic data, vital signs, laboratory test results, microbiological culture results, imaging data, treatment protocols, medication records, and survival information were recorded in MIMIC databases.

Due to the advantages of machine learning, we aim to develop machine learning models with AMI patients from Medical Information Mart for Intensive Care III and IV (MIMIC-III v1.4 and MIMIC-IV v1.0) databases to predict the risk of AKI.

## Materials and methods

### Data source

AMI patient data were extracted from the MIMIC-III v1.4 and MIMIC-IV v1.0 databases. The use of the MIMIC database was approved by the Institutional Review Board of the Beth Israel Deaconess Medical Center and Massachusetts Institute of Technology. We have obtained permission after application and completion of the course and test (record IDs: 44703031 and 44703032). Because all patient information in the database is anonymous, so informed consent was not required (23).

### Patients enrollment and data collection

SQL (Structured Query Language) programming in Navicat Premium (version 15.0.12) was used for data extraction. ICD-9 (International Classification of Diseases, Ninth Revision) codes were used to identify patients with AMI, and Codes 41000–41092 were used to identify the patients with AMI. Exclusion criteria: (1) Patients who are younger than 18 years or older than 90 years; (2) Patients with deficient test results of serum creatinine and troponin; (3) Patients with missing data of more than 5% were excluded from the analysis. (4) Patients admitted to the hospital for a recurrent episode of AMI. We randomly assigned 70% of the patients in the MIMIC-IV data to the training cohort, which is used to develop models in the training cohort. The remaining 30% is allocated to the testing cohort. Meanwhile, MIMIC-III patient data performs the external validation function of the model.

After identifying eligible subjects, we collected clinical data including demographics, comorbidities, vital signs, and laboratory parameters. Comorbidities include Atrial Fibrillation (AF), Heart Failure (HF), Diabetes Mellitus (DM), Hypercholesterolemia, Hypertriglyceridemia, Hypertension, Respiratory Failure, Ventricular Tachycardia (VT), and Cardiogenic Shock. Vital signs collect the first recorded results at the time of hospitalization, including heart rate, respiratory rate, body temperature, arterial systolic blood pressure, arterial diastolic blood pressure, and mean blood pressure. Laboratory parameters were also obtained for the first time after admission. The research indicators are red blood cells (RBC), white blood cells (WBC), platelets, hemoglobin, glucose, hematocrit, blood urea nitrogen (BUN), creatinine, potassium, sodium, chloride, calcium, phosphorus, magnesium, bicarbonate, activated partial prothrombin time (APTT), prothrombin time (PT), International Normalized Ratio (INR), Creatine Kinase Isozyme-MB (CK-MB), Troponin-T (TNT).

### Model construction and evaluation

Five machine learning models were constructed based on the features selected by the training cohort. The models used are: Decision Tree (DT) model, Support Vector Machine (SVM) model, Random Forest (RF) model, Naive Bayes (NB) model, and eXtreme Gradient Boosting (xGBoost) model. The 10-Fold cross-validation was used for model training. Among the five models, DT, SVM, RF, NB, and xGBoost are considered as the most common machine learning algorithms. DT (24) is very versatile machine learning model that can be used for both regression and classification. A decision tree is a tree-shaped structure in which each internal node represents a judgment on an attribute, each branch represents the output of a judgment result, and finally each leaf node represents a classification result. SVM (25) is a fast and dependable classification algorithm that performs very well with a limited amount of data. For classification, SVM works by creating a decision boundary in between our data points, that tries to separate it as best as possible. NB (26) is a model in a Bayesian classifier that trains a model with a dataset of known categories to achieve categorical judgment on data of unknown categories. The theoretical basis of NB is Bayesian decision theory. RF (27) is a kind of model that can be used both for regression and classification. It is one of the most popular ensemble methods, belonging to the specific category of bagging methods. This method can be described as techniques that use a group of weak learners together, in order to create a stronger, aggregated one. In our case, RF is an ensemble of many individual DT models. XGBoost (28) is an optimized distributed gradient boosting library designed to be highly efficient, flexible, and portable. It implements machine learning algorithms under the Gradient Boosting framework. The traditional logistic regression (LR) (29) model is also used for model construction. The nomogram (30) is used visualize regression models. and the calibration curve can be used as one of the evaluation indicators of the model. The calibration curve is used to evaluate the fit of the model (31). After the model is developed, data from the test cohort and validation cohort was used to further evaluate the performance of the model. The area under the receiver operating characteristic curve (AUC) and precision-recall curves was used to compare the performance of each model.

### Study endpoint

The study endpoint was AKI during hospitalization, which is based on a comprehensive assessment by the glomerular filtration rate to reflect renal function at admission and the changes of serum creatinine levels after admission. Estimating Glomerular filtration rate (eGFR) by Modification of Diet in Renal Disease (MDRD) study equation at admission was calculated from first serum creatinine level and age (32). The calculation formula was showed as following: (eGFR[mL/(min⋅1.73 m^2^) = 186⋅(*Scr*)^−1.154^⋅(age)^−0.203^). The diagnosis of AKI was based on the latest international AKI clinical practice guidelines (33). The diagnostic criteria are met in any of the following three criteria: (a) increase in creatinine by ≥ 0.3 mg/dl (≥26.5 μmol/l) within 48 h; (b) increase in creatinine to ≥ 1.5 times baseline, which is known or presumed to have occurred within the prior 7 days; (c) urine volume < 0.5 ml/kg/h for 6 h.

### Statistical analysis

In order to avoid excessive bias, the missing ratio of variables in this study was less than 5% and was imputed. Multiple imputation to account for missing data. The principle of multiple imputation was roughly divided into several points. First, several data sets containing all the missing variables were generated. Second, these datasets were used to build several complementary models, usually using generalized linear models. Third, these models were integrated together and then the performance of the multiple complementary models was evaluated. Finally, the complete dataset was output (34, 35).

Frequency and percentage were used to describe the categorical variables, and the chi-square test or Fisher’s exact test was used to identify differences between groups. The Shapiro-Wilk test was applied to continuous variables to confirm that they conformed to a normal distribution. All continuous variables in this study did not conform to a normal distribution and were described using the median and interquartile range (IQR), and the Mann–Whitney *U*-test was used to determine differences between different groups.

The training cohort consisted of 2,624 patients, including a heterogeneous sample of AKI and non-AKI patients, AKI patients accounted for only 29.4% of the entire cohort, whereas non-AKI patients accounted for 70.6% of the entire cohort. The proportions of these two categories are quite different, which may lead to lower prediction accuracy of the prediction model. Therefore, to solve the problem of classification imbalance, we used the synthetic minority oversampling technique (SMOTE) (36). The SMOTE method is an effective tool to solve the problem of data distribution imbalance. It is used in the training cohort to preprocess the data before the construction of the models.

The importance ranking of all variables was obtained by the SHapley Additive exPlanations (SHAP) method. SHAP could explain the output of any machine learning model. Its name came from the SHapley Additive exPlanation, inspired by cooperative game theory, SHAP constructed an additive explanatory model in which all features were considered as contributors. SHAP had a solid theoretical basis for achieving both local and global interpretability. The advantage of SHAP value was that it provided us not only SHAP values to evaluate feature importance, and it also showed us the positive or negative effects of the impact (37, 38).

R software (version 4.1.2) and Python software (version 3.10) were used for statistical analysis; GraphPad Prism (version 8.3.0) and Origin (version 9.1.0) was used to draw graphs; and *P < 0.05* was considered statistically significant.

## Results

### Baseline characteristics

**FIGURE 1:**
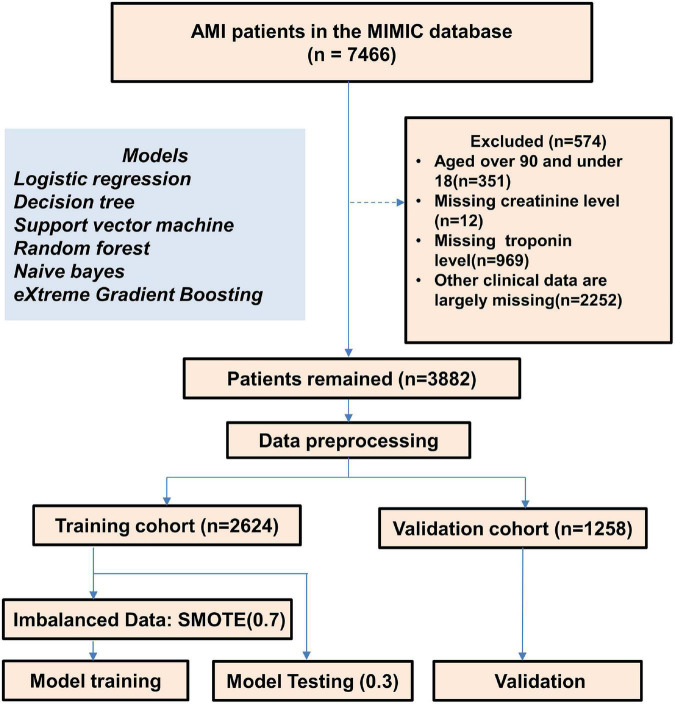
Flow diagram of the selection process of patients.

After applying the inclusion and exclusion criteria, 1,258 and 2,624 AMI patients were extracted from the MIMIC-III and MIMIC-IV database, respectively, and entered into the final analysis (Figure 1). In patients with AMI, the incidence of AKI was 25.8 and 29.4% in the MIMIC-III database and MIMIC-IV database, respectively. In the MIMIC-III group, there was no difference in the proportion of males in the AKI group and the non-acute kidney injury (non-AKI) group (*p* = 0.79), while the median age of the AKI group was significantly higher than that of the non-AKI group (*p* < 0.001). The proportion of males and median age in the AKI group were higher than those in the non-AKI group (*p* < 0.05, *p* < 0.001, respectively). Other baseline characteristics of the patients are shown in Table 1.

**TABLE 1:** Baseline characteristics.

	MIMIC III (*n* = 1,258)			MIMIC IV (*n* = 2,624)		
		
	Non-AKI (*n* = 933)	AKI (*n* = 325)	*P*-value	Non-AKI (*n* = 1,851)	AKI (*n* = 773)	*P*-value
Demographic						
Male (*n*%)	639 (68.5%)	220 (67.7%)	0.79	1,164 (62.9%)	518 (67.0%)	0.045
Age (year)	65.1 [55.3,75.4]	69.2 [58.7,78.4]	<0.001	67.0 [57.0,75.0]	71.0 [61.0,78.0]	<0.001
Vital signs						
Heart rate (min^–1^)	83.0 [72.0,95.0]	87.0 [73.0,100.0]	<0.05	83.0 [73.0,95.0]	86.0 [74.0,100]	<0.001
Temperature (°C)	36.5 [36.0,36.8]	36.5 [35.9,37.1]	0.548	36.6 [36.4,36.8]	36.6 [36.3,36.9]	0.789
Respiratory rate (min^–1^)	17.0 [15.0,21.0]	19.0 [15.0,22.0]	<0.05	18.0 [15.0,22.0]	19.0 [16.0,24.0]	<0.001
ASP (mmHg)	119.8 [106.0,134.0]	116.0 [101.0,133.0]	0.055	118.0 [105.0,134.0]	113 [101.0,131.0]	<0.001
ADP (mmHg)	61.0 [52.0,70.0]	59.0 [50.0,68.0]	<0.05	66.0 [56.0,76.0]	61.0 [53.0,72.0]	<0.001
MAP (mmHg)	78.0 [68.0,88.0]	75.0 [66.0,84.0]	<0.05	80.0 [71.0,91.0]	77.0 [69.0,87.0]	<0.001
Laboratory results						
RBC (m/uL)	4.2 [3.8,4.7]	3.9 [3.4,4.5]	<0.001	4.0 [3.4,4.5]	3.8 [3.2,4.3]	<0.001
WBC (k/uL)	11.0 [8.7,14.7]	12.2 [8.4,15.2]	0.088	10.5 [8.0,13.7]	11.1 [8.0,15.8]	<0.05
Platelet (k/uL)	239.0 [194.0,297.5]	222.0 [175.0,292.5]	<0.05	211.0 [165.0,265.0]	197.0 [153.5,250.0]	<0.001
Hemoglobin (g/dL)	13.0 [11.4,14.4]	12.1 [10.4,13.6]	<0.001	12.0 [10.1,13.7]	11.2 [9.7,13.0]	<0.001
Hematocrit (%)	37.8 [34.0,41.6]	36.0 [31.3,40.0]	<0.001	36.5 [31.1,40.6]	34.9 [30.2,39.5]	<0.001
Glucose (mg/dL)	140.0 [114.0,187.0]	155.0[118.0,230.5]	<0.001	133.0 [109.0,178.0]	148.0 [113.0,203.0]	<0.001
BUN (mg/dL)	18.0 [14.0,26.0]	25.0 [17.0,37.0]	<0.001	19.0 [14.0,32.0]	25.0 [18.5,39.0]	<0.001
Potassium (mEq/L)	4.1 [3.8,4.5]	4.3 [3.9,4.7]	<0.001	4.2 [3.9,4.5]	4.3 [3.9,4.6]	<0.05
Sodium (mEq/L)	139.0 [136.0,140.0]	138.0 [135.0,140.0]	0.083	138.0 [136.0,140.0]	138.0 [135.0,141.0]	0.858
Chloride (mEq/L)	103.0 [100.0,106.0]	102.0 [101.0,107.0]	0.353	103.0 [99.0,105.0]	103.0 [99.0,105.0]	0.613
Calcium (mg/dL)	8.6 [8.2,9.1]	8.4 [7.9,8.9]	<0.001	8.7 [8.2,9.1]	8.5 [8.0,9.0]	<0.001
Magnesium (mg/dL)	1.9 [1.7,2.1]	1.9 [1.7,2.1]	0.468	2.0 [1.8,2.1]	2.0 [1.8,2.2]	0.900
Phosphate (mg/dL)	3.4 [2.9,4.0]	3.6[3.0,4.5]	<0.001	3.6 [3.0,4.2]	3.8 [3.2,4.6]	<0.001
Bicarbonate (mEq/L)	23.0 [21.0,26.0]	23.0 [20.0,25.0]	<0.05	23.0 [21.0,25.0]	22.0 [19.0,25.0]	<0.001
APTT (s)	31.6 [26.1,55.4]	35.2 [27.6,59.2]	<0.05	35.6 [28.8,55.5]	39.4 [29.4,65.2]	<0.05
INR	1.2 [1.1,1.3]	1.2 [1.1,1.4]	<0.05	1.2 [1.1,1.3]	1.2 [1.1,1.4]	<0.001
PT (s)	13.4 [12.5,14.6]	13.5 [12.8,15.0]	<0.05	12.7 [11.7,14.6]	13.3 [12.1,15.8]	<0.001
CK-MB (ng/mL)	32.0 [8.0,94.5]	26.0 [8.0,97.0]	0.633	20.0 [6.0,71.0]	18.0 [5.0,69.1]	0.431
TNT (ng/mL)	1.0 [0.8,4.0]	1.5[0.2,5.5]	<0.05	0.5 [0.1,2.3]	0.5 [0.1,2.3]	0.978
Creatinine (mg/dL)	1.0[0.8,1.3]	1.3 [0.9,1.6]	<0.001	1.0 [0.8,1.5]	1.4 [1.0,1.9]	<0.001
GFR [mL/(min⋅1.73 m^2^)]	74.8 [55.8,95.8]	56.3 [38.1,82.7]	<0.001	72.0 [44.7,99.3]	52.1 [33.5,75.9]	<0.001
Comorbidities (*n*%)						
HF (*n*%)	327 (35.0%)	154 (47.4%)	<0.001	218 (11.8%)	100 (12.9%)	0.407
Cardiogenic shock (*n*%)	132 (14.1%)	85 (26.2%)	<0.001	85 (4.6%)	82 (10.4%)	<0.001
Atrial fibrillation (*n*%)	189 (19.9%)	110 (33.8%)	<0.001	501 (27.1%)	325 (42.0%)	<0.001
Hypertension (*n*%)	467 (50.1%)	144 (44.3%)	0.074	339 (18.3%)	96 (12.4%)	<0.001
Hyperlipidemia (*n*%)	273 (29.3%)	77(23.7%)	0.054	1,003 (54.2%)	339 (51.6%)	0.229
Hypercholesterolemia (*n*%)	154 (16.5%)	34 (10.5%)	<0.05	90 (4.9%)	39 (5.0%)	0.843
Respiratory failure (*n*%)	107 (11.5%)	72 (22.2%)	<0.001	70 (3.8%)	39 (5.0%)	0.162
DM (*n*%)	188 (20.2%)	86 (26.5%)	0.018	171 (9.2%)	77 (10.0%)	0.559
Ventricular tachycardia (*n*%)	119 (12.8%)	47 (14.5%)	0.434	90 (4.9%)	73 (9.4)	<0.001

Continuous variables are presented as the median and interquartile range (IQR). Counting data are presented as numbers and percentages. ASP, arterial systolic pressure; ADP, diastolic arterial pressure; MAP, mean arterial pressure; RBC, red blood cell; WBC, white blood cell; BUN, blood urea nitrogen; APTT, activated partial prothrombin time; INR, International Normalized Ratio; PT, prothrombin time; CK-MB, Creatine Kinase Isozyme-MB; TNT, Troponin-T; GFR, Glomerular Filtration Rate; HF, Heart Failure; DM, Diabetes Mellitus.

### Feature selection for models

**FIGURE 2:**
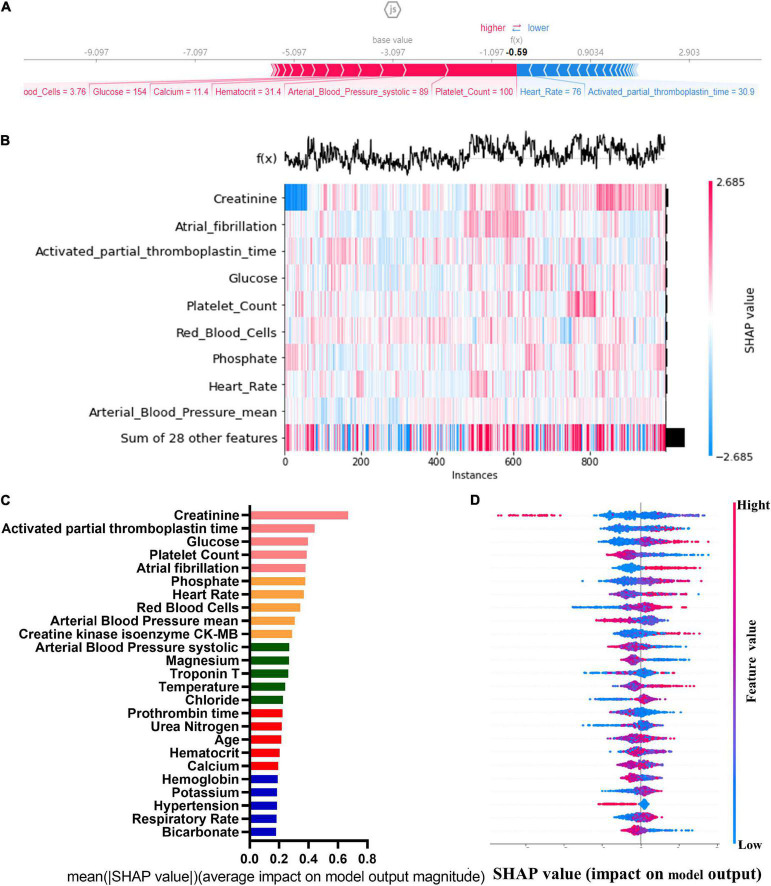
Single-sample feature impact map **(A)**; heat map of feature distribution under sample clustering **(B)**; histogram of feature importance **(C)**; scatter plot of feature density **(D)**.

The SHAP graph group are shown in Figure 2, including single-sample feature influence map, feature distribution heat map under sample clustering, feature importance histogram, and feature density scatter plot. SHAP gives variables importance ranking, which relies on the xGBoost classification algorithm, and provides an intrinsic measure of the importance of each feature, called the Shap value (39). The top 5 predictors were serum creatinine, activated partial prothrombin time, blood glucose concentration, platelets, and atrial fibrillation (SHAP values are 0.670, 0.444, 0.398, 0.389, and 0.381, respectively). We then developed machine learning models which included top 5 variables, top 10 variables, top 15 variables, top 20 variables, top 25 variables, and all variables, respectively, according to the variable importance ranking.

### Logistic regression model

**FIGURE 3:**
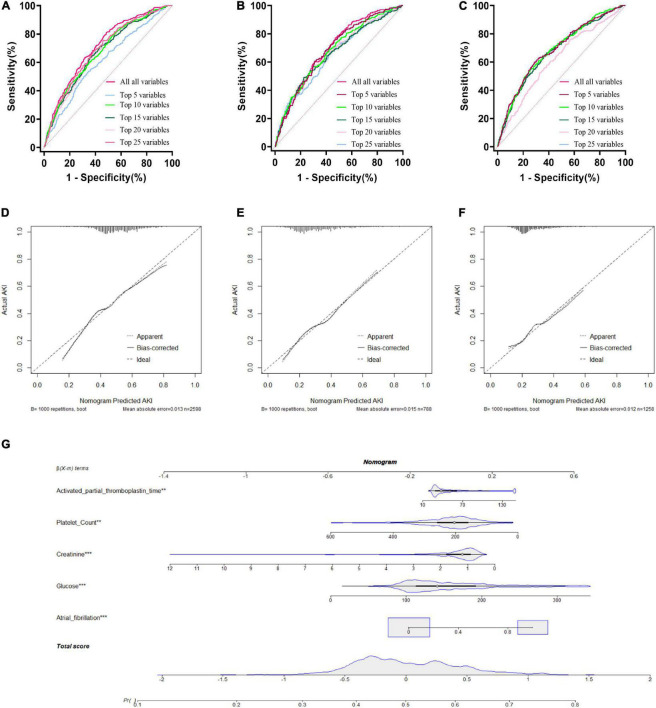
Logistic Regression model with different variables ROC curves; training cohort **(A)**; test cohort **(B)**; validation cohort **(C)**. Logistic Regression model calibration curve; training cohort **(D)**; test cohort **(E)**; validation cohort **(F)**. Logistic Regression model Nomogram **(G)**.

**FIGURE 4:**
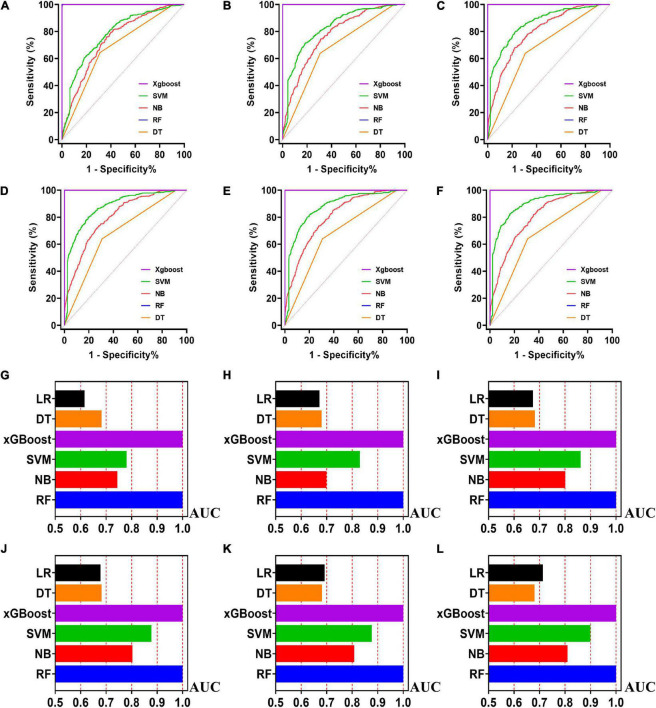
The ROC curves for machine learning models and the performances of all models in test cohort. The X-axis in 4G-4L represents the AUC values of each model. Top 5 variables **(A,G)**; top 10 variables **(B,H)**; top 15 variables **(C,I)**; top 20 variables **(D,J)**; top 25 variables **(E,K)**; models for all variables **(F,L)**.

**FIGURE 5:**
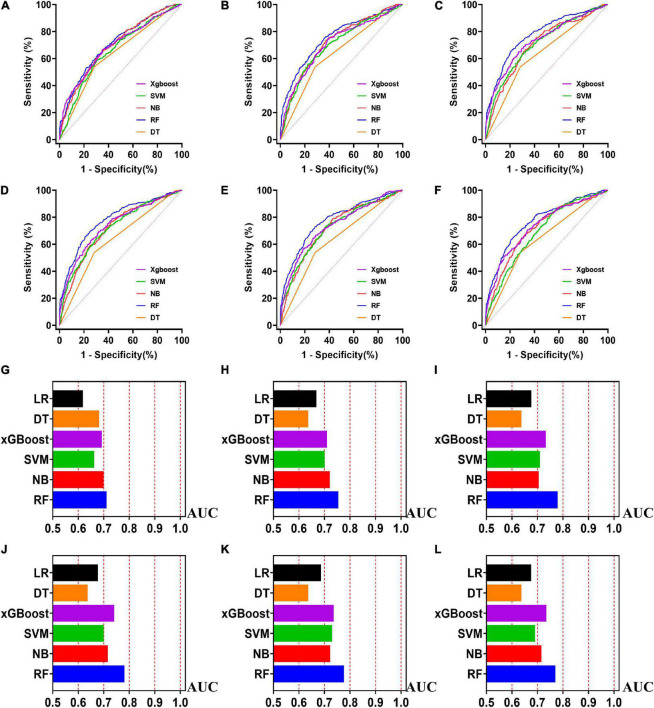
The ROC curves for machine learning models and the performances of all models in validation cohort. The X-axis in 5G-5L represents the AUC values of each model. Top 5 variables **(A,G)**; top 10 variables **(B,H)**; top 15 variables **(C,I)**; top 20 variables **(D,J)**; top 25 variables **(E,K)**; models for all variables **(F,L)**.

A LR model was first developed that included the top 5 most important variables, creatinine, activated partial prothrombin time, glucose, platelets, and atrial fibrillation. The LR model was plotted the receiver operating characteristic (ROC) curve (Figure 3) in the training cohort, and the AUC was calculated to be 0.615 (Figure 4). Meanwhile, the Nomogram and the Calibration curves are shown in the training cohort, test cohort, and validation cohort (Figure 3). The LR model with all variables (LR-all) in training cohort achieved an AUC of 0.713, (95% CI: 0.693∼0.732) (Figure 4). Meanwhile, the LR-all model in test cohort, achieved an AUC of 0.694 (95% CI: 0.656∼0.733) (Supplementary Figure 1). In the validation cohort, the AUC of the LR model with top 20 variables (LR-20) performed best in validation cohort (with an AUC of 0.686, 95%CI: 0.653∼0.720) (Figure 5).

### Machine learning models in the training cohort

**FIGURE 6:**
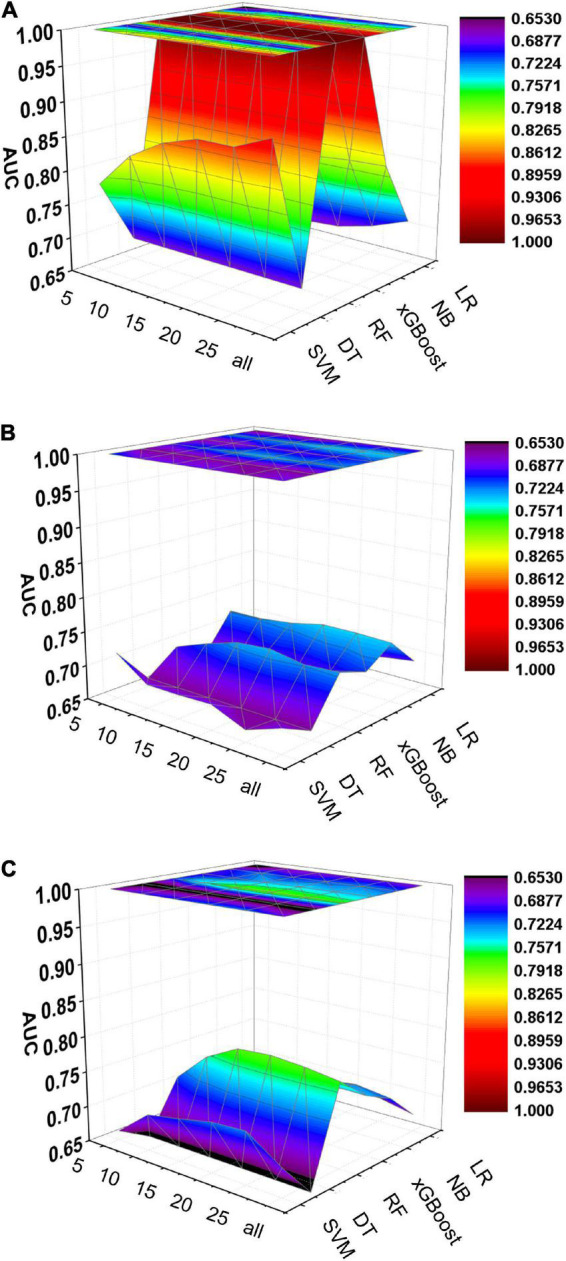
Dynamic plot of the area under the ROC curve for all machine learning models; training cohort **(A)**; test cohort **(B)**; validation cohort **(C)**. The x-axis represents the number of variables included in the model, the y-axis represents different kinds of models, and the z-axis represents the AUC values of each model.

Five machine learning models including RF, NB, SVM, xGBoost, DT were then developed. According to the order of variable importance, top 5 variables, top 10 variables, top 15 variables, top 20 variables, top 25 variables and models including all variables were successively developed. The machine learning models of using top 5 important features in training cohort were as follows: the RF model (RF-5), with an AUC of 1 (95% CI: 1); the NB model (NB-5), with an AUC of 0.744, (95% CI: 0.725∼0.763); the SVM model (SVM-5), with an AUC of 0.750 (95% CI: 0.730∼0.769); the xGBoost model (xGBoost-5), with an AUC of 1 (95% CI: 1); the DT model (DT-5), with an AUC of 0.682 (95% CI: 0.662∼0.703). All machine learning models outperformed the LR model in the training cohort. The RF-5 model and xGBoost-5 performed the best of all machine learning models. The DT-5 model has the worst performance. NB-5 and SVM-5 perform well in the training cohort. The SVM-5 model outperforms the NB-5 model. We gradually increased the number of included variables to develop different machine learning models. The ROC curves are shown for each model in the training cohort (Figure 4). The performances of all of the models are shown in the training cohort (Figure 4), and statistics for all models in the training cohort are shown in Supplementary Tables 1–6. There are, as the number of variables increased, dynamic plot of the area under the ROC curve for all machine learning models in training cohort (Figure 6).

### Machine learning models in the test cohort

The other 30% of the data in MIMIC-IV is used as a test cohort to test the performance of each machine learning model. The machine learning models of the variable importance top 5 in test cohort were as follows: the RF-5 model, with an AUC of 0.696 (95% CI: 0.658∼0.734); the NB-5 model, with an AUC of 0.724 (95% CI: 0.687∼0.762); the SVM-5 model, with an AUC of 0.718 (95% CI: 0.680∼0.756); the xGBoost-5 model, with an AUC of 0.666 (95% CI: 0.626∼0.706); the DT-5 model, with an AUC of 0.663 (95% CI: 0.622∼0.704). The performance of the five machine learning models in the test cohort is better than that of the NB model, and the AUC of LR model with top 5 variables (LR-5) has only 0.652 in the test cohort. The NB model is the best performer of all machine learning models in the test cohort. The NB model performed best when the variable importance top 20 variables were added to the model (AUC for NB-20 model: 0.739, 95% CI: 0.702∼0.776). The worst performing model is DT model with top 20 variables (AUC for DT-20 model: 0.663, 95% CI: 0.622∼0.704). xGBoost model performed general in the test cohort and their AUC increased when more variables were added (AUC for xGBoost-20 model: 0.689, 95% CI: 0.650∼0.728). But, the AUC of SVM model not increased when more variables were added (SVM-20 model: 0.687, 95% CI: 0.647∼0.727). And outperformed the RF-20 model (AUC for RF-20 model: 0.733, 95% CI: 0.695∼0.770). The ROC curves are shown for each model (Supplementary Figure 1). The performances of all models are shown in the test cohort (Supplementary Figure 1). Statistical measures of the performance of all models in the test cohort are shown in Supplementary Tables 7–12. There are, as the number of variables increased, dynamic plot of the area under the ROC curve for all machine learning models in test cohort (Figure 6).

### Machine learning models in the validation cohort

**FIGURE 7:**
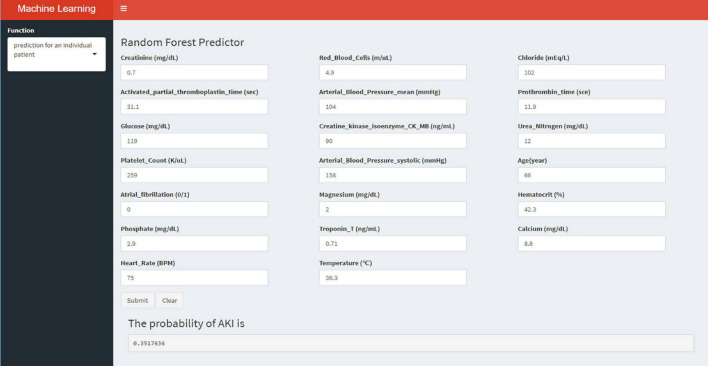
An example of the application software for predicting AKI risk in AMI patients.

Externally validated in a validation cohort of 1,258 cases, among all developed machine learning models, the RF model performed the best, followed by xGBoost, and the worst performing model was DT. The RF models were as follows in the external validation cohort: the RF-5 model, with an AUC of 0.711 (95% CI: 0.678∼0.744); the RF model with top 10 variables (RF-10), with an AUC of 0.754 (95% CI: 0.722∼0.786); the RF model with top 15 variables (RF-15), with an AUC of 0.778, (95% CI: 0.747∼0.808); the RF-20 model, with an AUC of 0.781, (95% CI: 0.750∼0.811); the RF model with top 25 variables (RF-25), with an AUC, 0.777 (95% CI: 0.746∼0.807); the RF model with all variables (RF-all), with an AUC of 0.770 (95% CI: 0.740∼0.801). The AUC of DT model with all variables (DT-all) in the validation cohort was 0.637 (95% CI: 0.602∼0.672). The AUC of LR-all was 0.686 (95% CI: 0.653∼0.720) in the validation cohort. The ROC curves are shown for each model (Figure 5). The performances of all models are shown in the validation cohort (Figure 5), and statistical measures of the performance for the variable importance top20 models in the validation cohort (Table 2). Statistical measures of performance of other models are shown in the validation (Supplementary Tables 13–17). Meanwhile, there are, as the number of variables increased, dynamic plot of the area under the ROC curve for all machine learning models in validation cohort (Figure 6). Precision-recall curves of the six models with different variables in the validation cohort were showed in Supplementary Figures 2–7. The area under the precision-recall curves of each model was calculated. Consistent with the AUC values, it indicated that random forest outperformed the other five models in the validation cohort. Moreover, an application software program based on the top 20 predictors were developed for evaluating the risk of AKI. The AKI probability of each patient could be calculated after the patient was admitted to hospital (Figure 7).

**TABLE 2:** The performance of six models containing the top 20 importance variables.

Model	AUC (CI 95%)	Accuracy	Sensitivity	Specificity
Support Vector Machine	0.720 (0.687∼0.753)	0.684	0.697	0.646
Decision Trees	0.637 (0.602∼0.672)	0.670	0.716	0.538
Random Forests	0.781 (0.750∼0.811)	0.735	0.748	0.698
eXtreme Gradient Boosting	0.741 (0.708∼0.773)	0.682	0.678	0.695
Naive Bayes	0.716 (0.684∼0.749	0.628	0.582	0.763
Logistic regression	0.686 (0.653∼0.720)	0.694	0.743	0.550

## Discussion

This study identified various clinical features associated with the risk of AKI in patients with AMI. Using a sophisticated machine learning approach, we found that creatinine, blood urea nitrogen, atrial fibrillation, glucose and hemoglobin were considered as the most important five features with AKI in patients with AMI. Among the six models, the RF model has the best performance with an AUC of 0.781 for the RF-20 model in the external validation cohort. The results of this study showed that the occurrence of AKI in patients with AMI was 28.2%. Compared with previously reported studies, the incidence of in-hospital AKI in patients with AMI reported in this study is close to the upper limit of normal (2–4). The possible reasons are follows; (a) firstly, we excluded patients with more than 5% missing data, which resulted in fewer hospitalizations for AMI overall and ultimately led to a higher incidence of AKI; (b) secondly, the median age of patients in each group was high (>65 years) for both MIMIC-III and MIMIC-IV, which suggest that general condition of our study population is not very optimistic, and they have poor resistance to injury.

Early identification of AKI in patients admitted for AMI improves overall outcomes (19). Therefore, identifying risk factors for AKI in patients with AMI can help to identify high-risk patients and to make appropriate clinical decisions. With the development of machine learning algorithms, the number of predictors that can be processed has largely been enriched. Therefore, advanced machine learning techniques allow researchers to develop more optimized models compared to traditional models (40). With such a model, cardiologists can be alerted in advance when a patient is admitted to the hospital with an AMI.

Zhou et al. reported a risk model for AKI prediction in AMI patients with LR analysis. The model calibrated well and performed better than traditional risk scores (41). With the development of concepts such as real-world research and precision treatment, the demand for medical big data processing by scientific researchers is increasing. Machine learning technology had a unique advantage in processing massive and high-dimensional data and conducting predictive evaluation, so in recent years, the application of machine learning method in the medical field had been deepening. Sun et al. developed several machine learning models and found that random forest model out performed outperformed LR in every comparison (36). Random forest methods improved the accuracy of AKI risk stratifying in AMI patients. The sample size of this study was relatively small and this was a single-center study, so we tried to explore a more robust AKI risk prediction model with a larger sample size from another canter. This study is an example of how machine learning methods works for evaluating AKI risk in AMI patients. Similarly, machine learning algorithms can be applied to other risk assessments of AMI patients, such as the risk of all-cause mortality, the risk of cardiac mortality and the incidence of major adverse cardiac event (MACE). The association between the risk factors and the risk of AKI is established by using artificial intelligence, and indicators such as patients’ vital signs and laboratory test results are matched with AKI risks, which helps to improve the risk perception and recognition ability of the model. This innovative approach to risk assessment helps clinicians benefit from better individualized treatment decisions.

In the present study, we used advanced statistical methods and specially processed data. The former includes five machine learning algorithm development models and traditional LR development models, with the 70% subset used for training cohort, and the 30% subset used for internal testing. Meanwhile, the data in MIMIC-III were used external validation and the ROCs to evaluate the models (28). Although there are many ways to filter the importance of variables, such as Boruta Algorithm and LASSO Regression (36, 42), SHAP method was used in the present study for feature selection. SHAP method not only shows the contribution of all features to the model output at the macro level with the feature density scatter plot, feature importance SHAP value and feature distribution heat map under sample clustering, but also shows the model output at the micro level through a single sample feature influence map (43, 44). Machine learning techniques help doctors analyze large amounts of information and are critical in optimizing medical practice. The latter is that we used the data in MIMIC-IV, the training and test cohort, to create a new dataset with a 1:1 ratio of AKI to non-AKI, addressing the imbalance of samples.

MIMIC, a high-quality database with a large sample size, was used in this study. There are several advantages for using the database. (a) Firstly, it is one of the few critical care databases that is freely accessible. (b) Secondly, the dataset spans more than a decade and contains a wealth of detailed information on patient care. (c) Thirdly, once the data usage agreement is accepted, the investigator’s analysis is not subject to limitations, thereby enabling clinical research and education around the world. (d) Finally, data can be downloaded from multiple sources (45).

There are also some limitations in our study. Firstly, our model was developed retrospectively based on a single-center database. Missing data and input errors exist, such as C-reactive protein and N-terminal pro-brain natriuretic peptide, despite the very high quality of the MIMIC databases. Therefore, prospective validation of our model in another cohort is still required in the future (46, 47). Secondly, we trained the model and tested it using synthetic datasets due to the severe class imbalance of the extracted datasets, which could have led to, in the training cohort, over fitting of models cannot be avoided and an overly optimistic assessment of its performance (48). Thirdly, this study only focused on the incidence of AKI during hospitalization, while other important prognostic indicators such as long-term mortality after discharge still require further investigation.

## Conclusion

We have developed several machine learning prediction models based on the MIMIC database. Among them, the RF model has good performance and can be used to guide clinical practice.

## Data availability statement

The datasets presented in this study can be found in online repositories. The names of the repository/repositories and accession number(s) can be found below: https://physionet.org/content/mimiciii-demo/1.4/ and https://physionet.org/content/mimiciv/1.0/.

## Ethics statement

The studies involving human participants were reviewed and approved by the Beth Israel Women’s Deaconess Medical Center and the MIT Institutional Review Board. The ethics committee waived the requirement of written informed consent for participation.

## Author contributions

DC analyzed the data and wrote the manuscript. TX and YW collect the data. LM, AZ, and BC checked the integrity of the data and the accuracy of the data analysis. LS, QW, and YJ co-designed and revised the article. All authors read and approved the final manuscript.
