# Supplementary material for: Predicting acute kidney injury risk in acute myocardial infarction patients: An artificial intelligence model using medical information mart for intensive care databases
Source: Front Cardiovasc Med. 2022 Sep 7;9:964894. doi: 10.3389/fcvm.2022.964894 (PMC9489917; doi:10.3389/fcvm.2022.964894)
Supplement: Supplementary file 1 [file Data_Sheet_1.PDF]

Supplementary Figure 1: The ROC curves for machine learning models and the performances of all models in the test cohort. Top 5 variables (A, G); top 10 variables (B, H); top 15 variables (C, I); top 20variables (D, J); top 25 variables (E, K); Models for all variables (F, L).

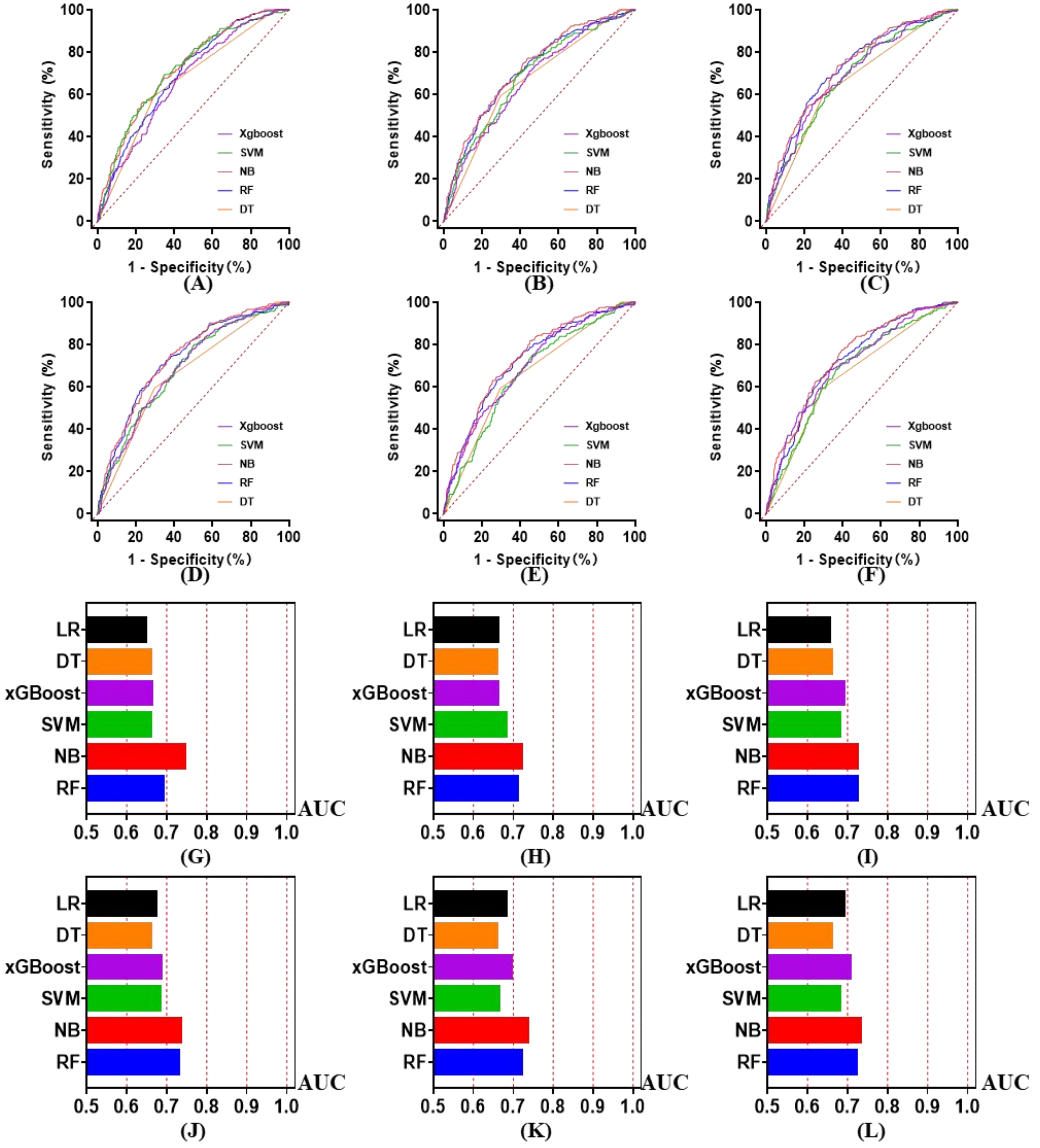

Supplementary Figure 2: Precision-recall curves of LR models with different variables in the training cohort. Top 5 variables (A); top 10 variables (B); top 15 variables (C); top 20 variables (D); top 25 variables (E); Models for all variables (F).

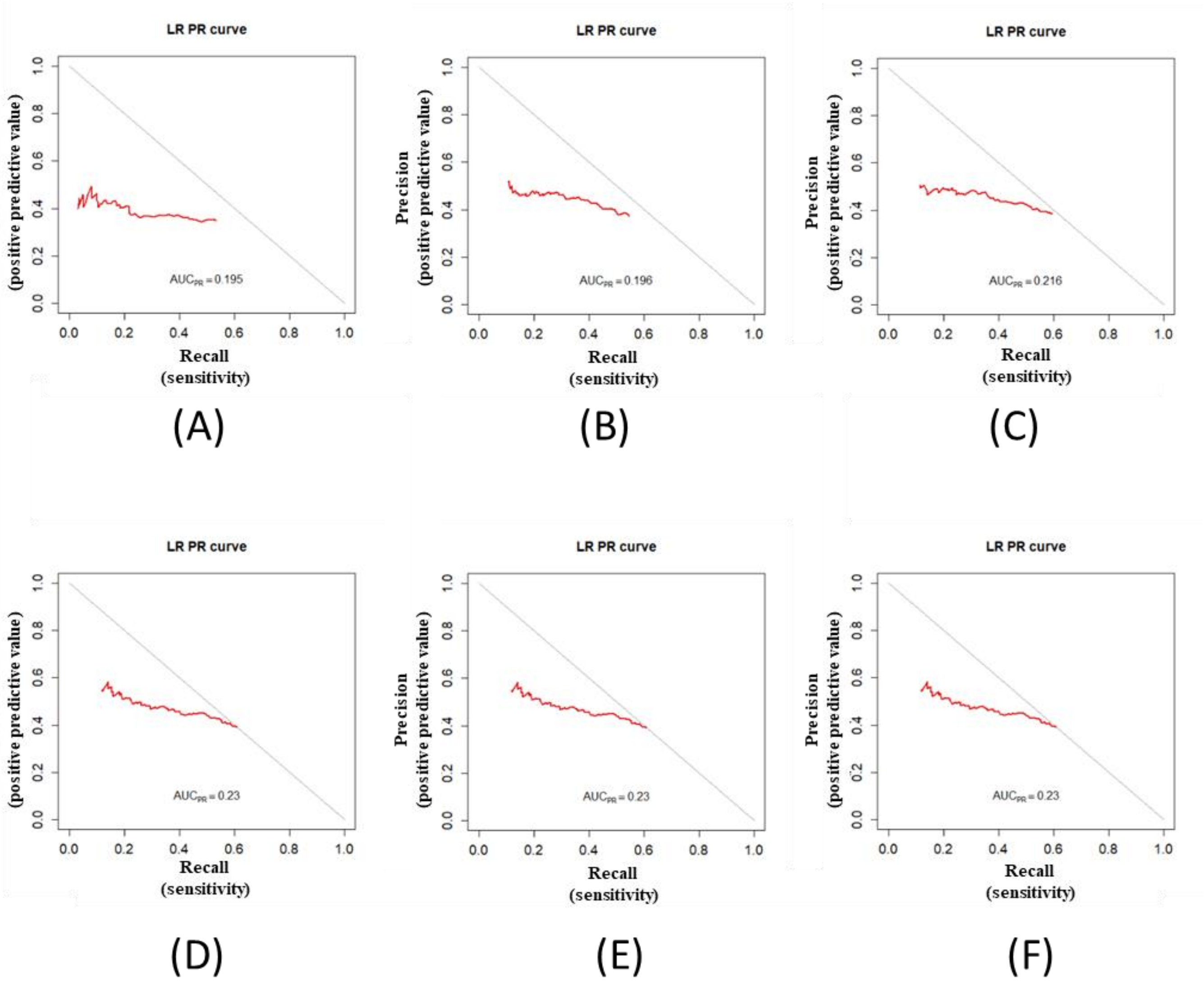

Supplementary Figure 3: Precision-recall curves of DT models with different variables in the training cohort. Top 5 variables (A); top 10 variables (B); top 15 variables (C); top 20 variables (D); top 25 variables (E); Models for all variables (F).

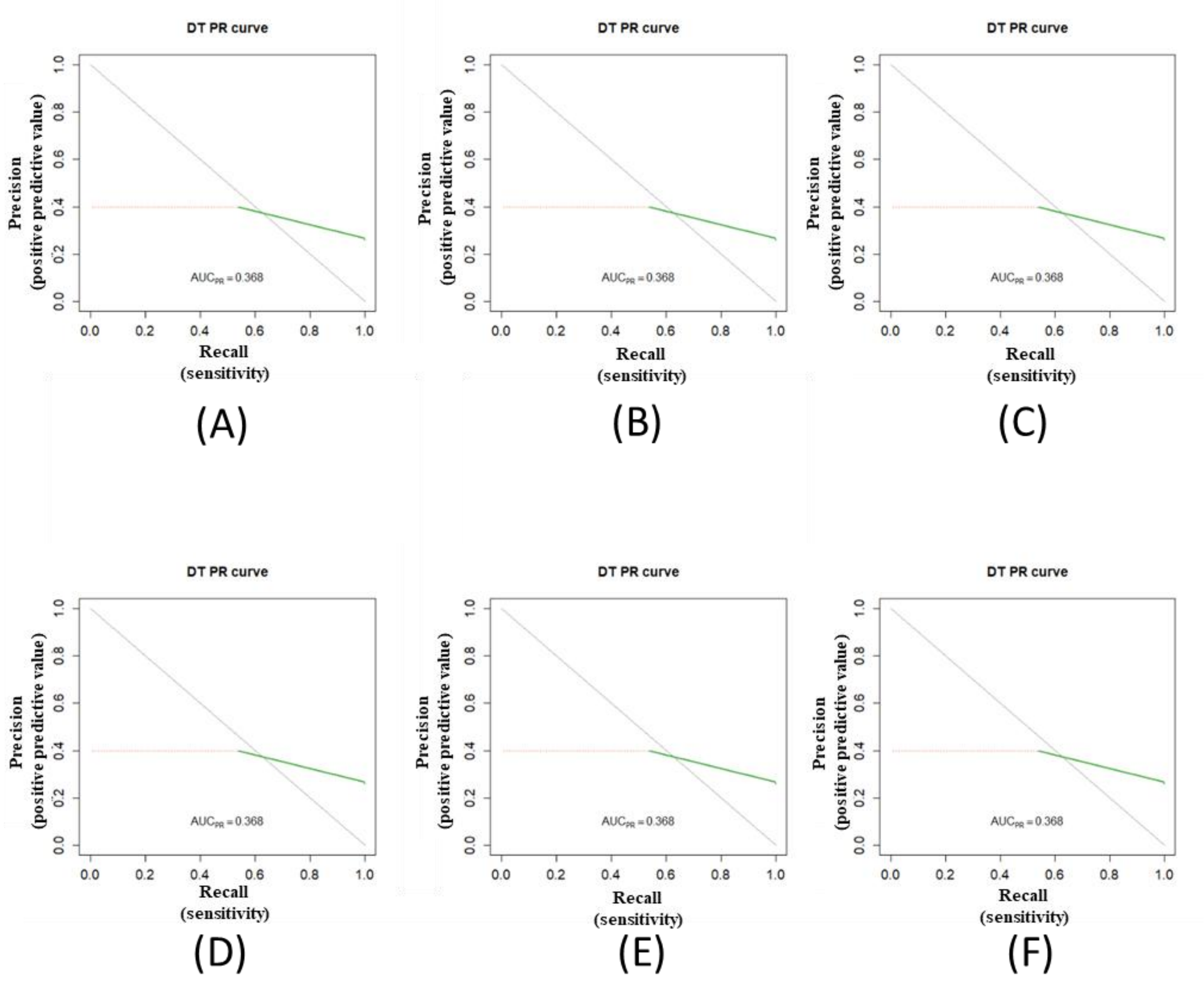

Supplementary Figure 4: Precision-recall curves of RF models with different variables in the training cohort. Top 5 variables (A); top 10 variables (B); top 15 variables (C); top 20 variables (D); top 25 variables (E); Models for all variables (F).

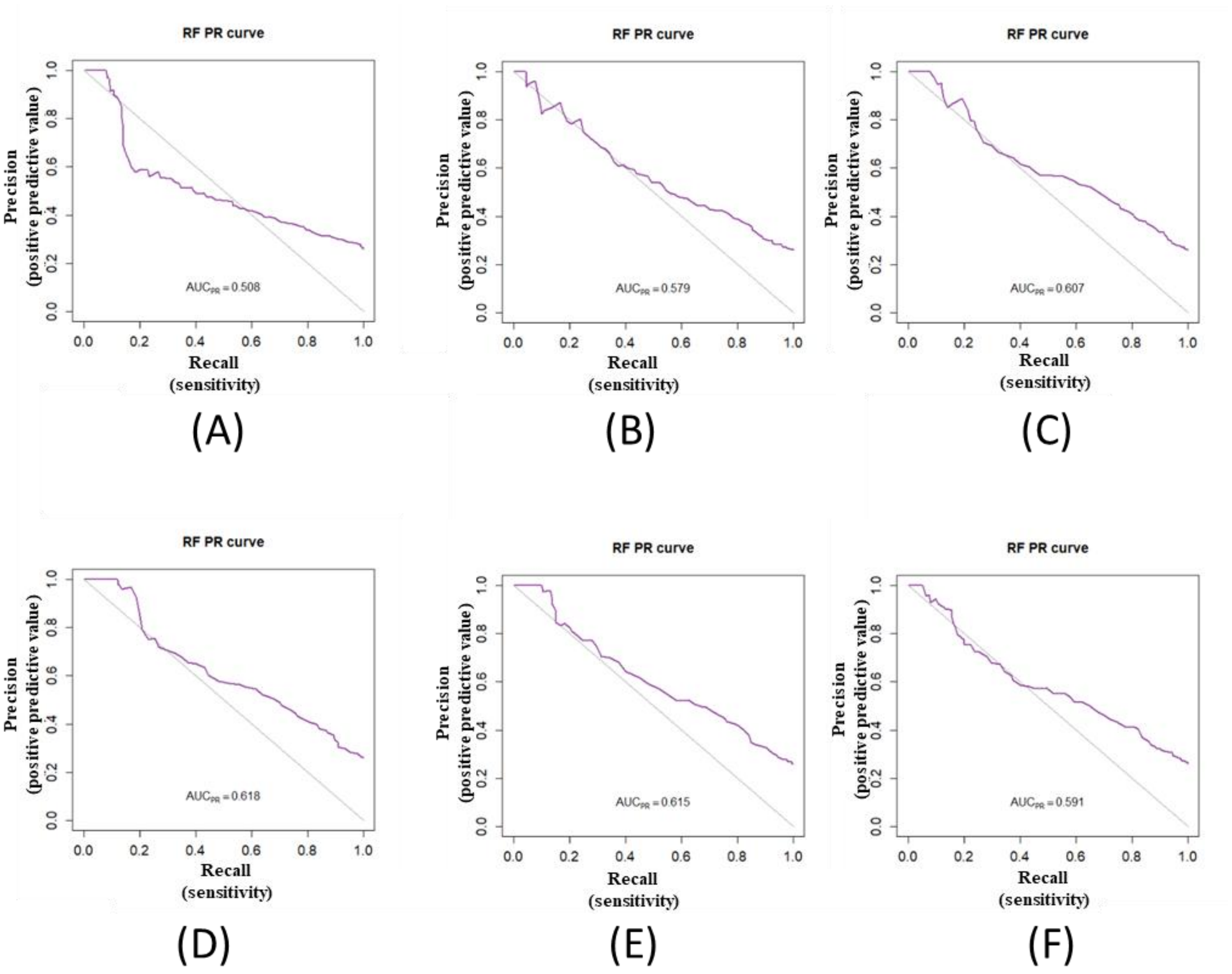

Supplementary Figure 5: Precision-recall curves of xGBoost models with different variables in the training cohort. Top 5 variables (A); top 10 variables (B); top 15 variables (C); top 20 variables (D); top 25 variables (E); Models for all variables (F).

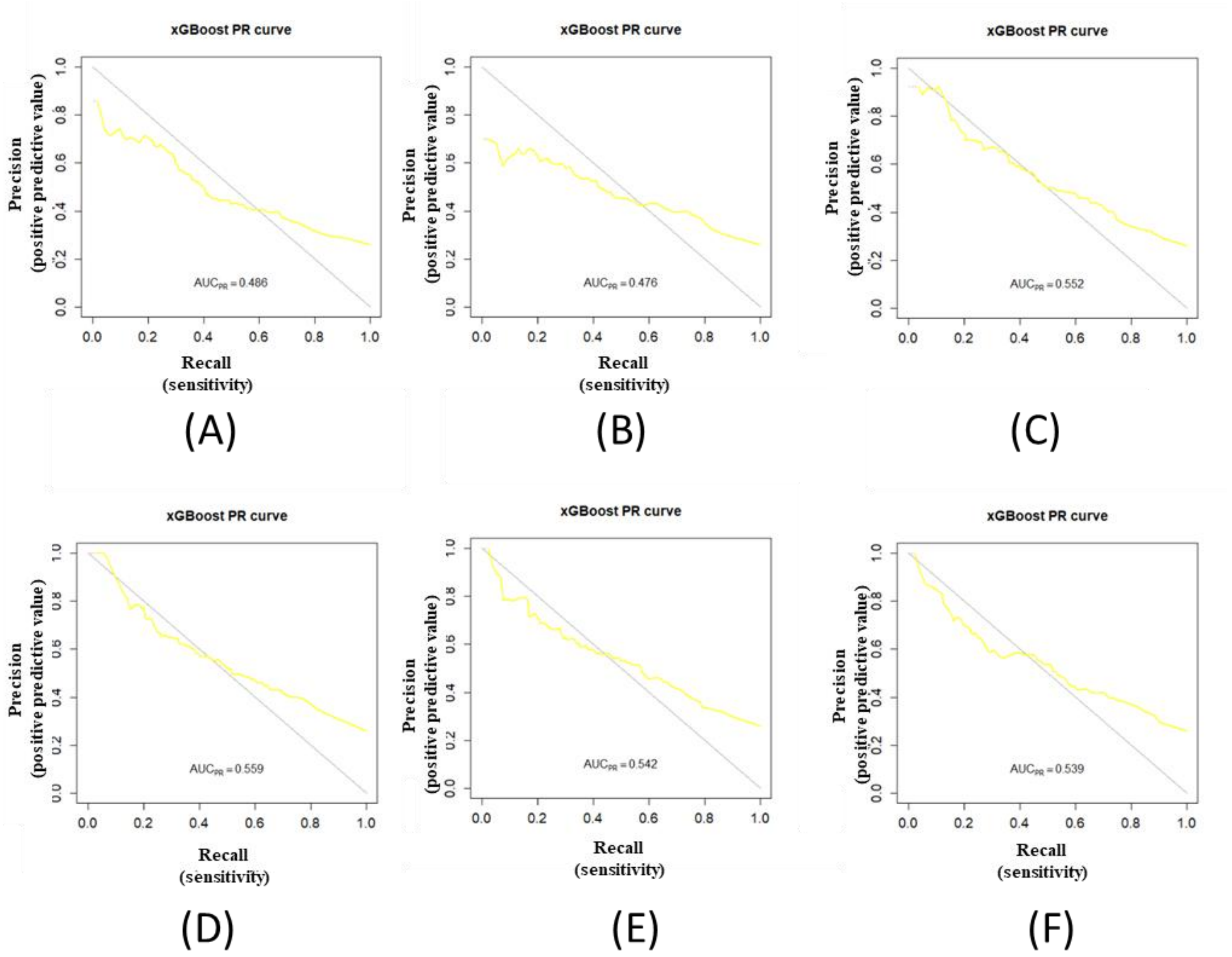

Supplementary Figure 6: Precision-recall curves of NB models with different variables in the training cohort. Top 5 variables (A); top 10 variables (B); top 15 variables (C); top 20 variables (D); top 25 variables (E); Models for all variables (F).

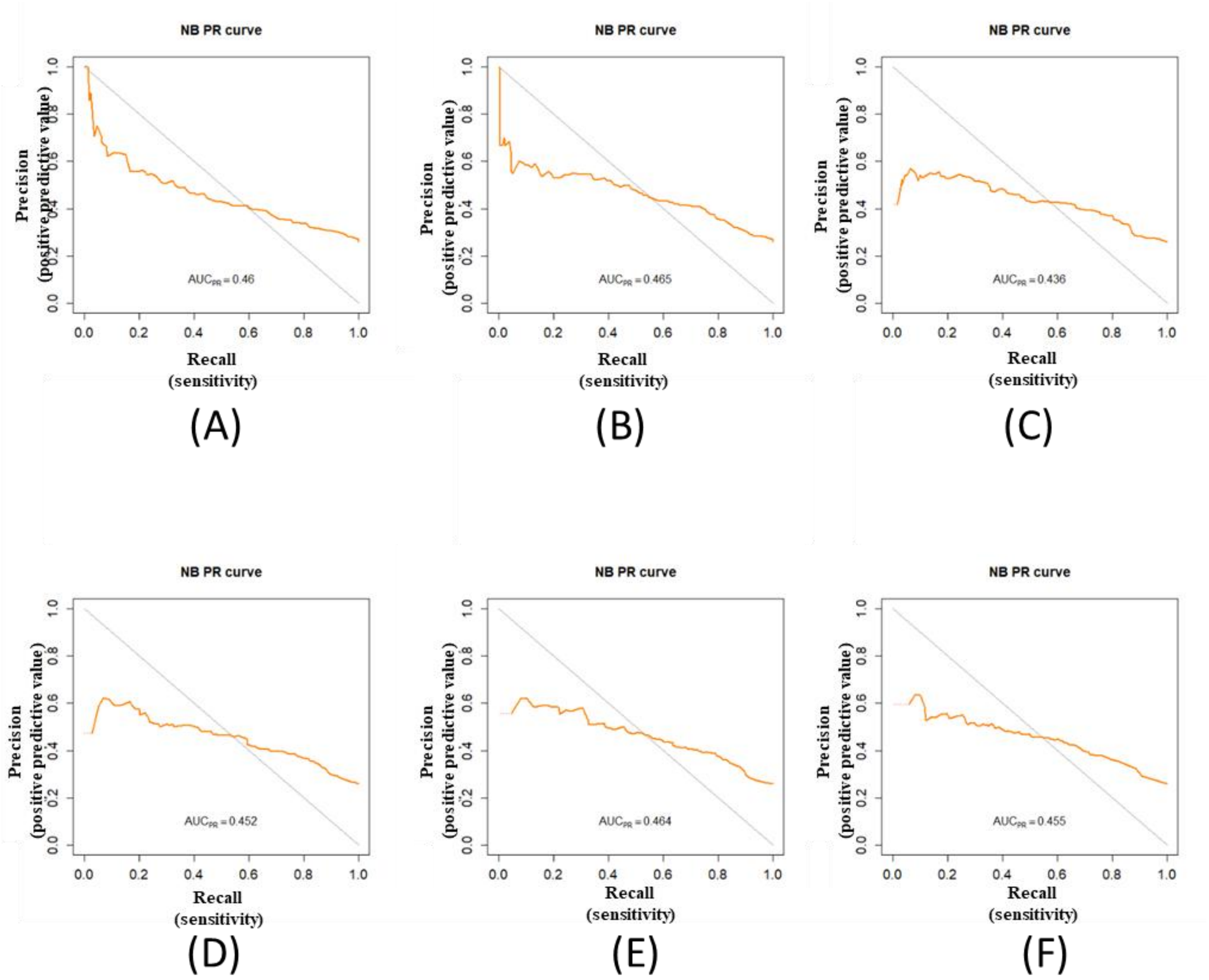

Supplementary Figure 7: Precision-recall curves of SVM models with different variables in the training cohort. Top 5 variables (A); top 10 variables (B); top 15 variables (C); top 20 variables (D); top 25 variables (E); Models for all variables (F).

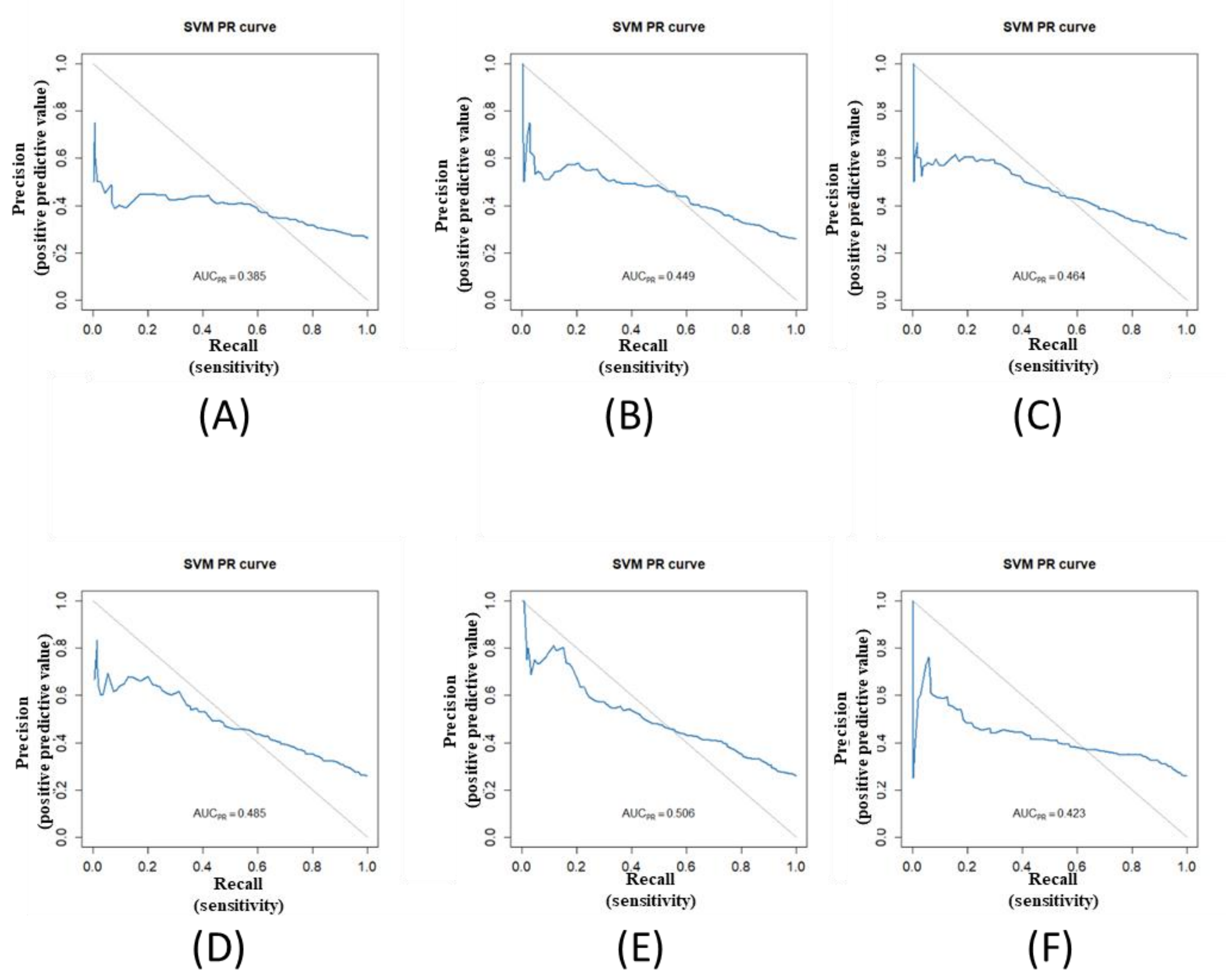

Supplementary table 1. Statistical measures of performance of the models in the training cohort; top 5 variables.

Model	AUC (CI 95%)	Accuracy	Sensitivity	Specificity
SVM	0.750(0.730 ~ 0.769)	0.710	0.610	0.811
DT	0.682(0.662 ~ 0.703)	0.666	0.693	0.639
RF	1(1.000)	1.000	1.000	1.000
xGBoost	1(1.000)	1.000	1.000	1.000
NB	0.744(0.725 ~ 0.763)	0.698	0.604	0.792
LR	0.615(0.593 ~ 0.636)	0.595	0.652	0.538

Supplementary table 2. Statistical measures of performance of the models in the training cohort; top 10 variables.

Model	AUC (CI 95%)	Accuracy	Sensitivity	Specificity
SVM	0.786(0.767 ~ 0.804)	0.764	0.814	0.715
DT	0.682(0.662 ~ 0.703)	0.666	0.693	0.639
RF	1(1.000)	1.000	1.000	1.000
xGBoost	1(1.000)	1.000	1.000	1.000
NB	0.778(0.760 ~ 0.795)	0.715	0.686	0.744
LR	0.672(0.651 ~ 0.692)	0.635	0.649	0.620

Supplementary table 3. Statistical measures of performance of the models in the training cohort; top 15 variables.

Model	AUC (CI 95%)	Accuracy	Sensitivity	Specificity
SVM	0.800(0.782 ~ 0.818)	0.787	0.779	0.795
DT	0.682(0.662 ~ 0.703)	0.666	0.693	0.639
RF	1(1.000)	1.000	1.000	1.000
xGBoost	1(1.000)	1.000	1.000	1.000
NB	0.801(0.785 ~ 0.818)	0.780	0.679	0.778
LR	0.674(0.653 ~ 0.694)	0.639	0.566	0.711

Supplementary table 4. Statistical measures of performance of the modesl in the training cohort; top 20 variables.

Model	AUC (CI 95%)	Accuracy	Sensitivity	Specificity
SVM	0.827(0.810 ~ 0.844)	0.804	0.806	0.802
DT	0.682(0.682)	0.666	0.693	0.639
RF	1(1.000)	1.000	1.000	1.000
xGBoost	1(1.000)	1.000	1.000	1.000
NB	0.803(0.786 ~ 0.819)	0.727	0.708	0.746
LR	0.678(0.658 ~ 0.699)	0.628	0.473	0.783

Supplementary table 5. Statistical measures of performance of the models in the training cohort; top 25 variables.

Model	AUC (CI 95%)	Accuracy	Sensitivity	Specificity
SVM	0.830(0.813 ~ 0.847)	0.809	0.816	0.802
DT	0.682(0.662 ~ 0.703)	0.666	0.693	0.639
RF	1(1.000)	1.000	1.000	1.000
xGBoost	1(1.000)	1.000	1.000	1.000
NB	0.808(0.792 ~ 0.824)	0.729	0.605	0.852
LR	0.692(0.672 ~ 0.712)	0.646	0.618	0.675

Supplementary table 6. Statistical measures of performance of the models in the training cohort; models for all variables.

Model	AUC (CI 95%)	Accuracy	Sensitivity	Specificity
SVM	0.844(0.827 ~ 0.860)	0.832	0.836	0.828
DT	0.682(0.662 ~ 0.703)	0.663	0.693	0.639
RF	1(1.000)	1.000	1.000	1.000
xGBoost	1(1.000)	1.000	1.000	1.000
NB	0.810(0.793 ~ 0.826)	0.732	0.646	0.819
LR	0.713(0.693 ~ 0.732)	0.662	0.513	0.812

Supplementary table 7. Statistical measures of performance of the models in the test cohort. Top5 variables.

Model	AUC (CI 95%)	Accuracy	Sensitivity	Specificity
SVM	0.718(0.680 ~ 0.756)	0.666	0.655	0.690
DT	0.663(0.622 ~ 0.704)	0.671	0.704	0.593
RF	0.696(0.658 ~ 0.734)	0.610	0.543	0.766
xGBoost	0.666(0.626 ~ 0.706)	0.615	0.585	0.686
NB	0.724(0.687 ~ 0.762)	0.661	0.654	0.678
LR	0.652(0.610 ~ 0.694)	0.599	0.570	0.665

Supplementary table 8. Statistical measures of performance of the models in the test cohort; top 10 variables.

Model	AUC (CI 95%)	Accuracy	Sensitivity	Specificity
SVM	0.685(0.645 ~ 0.725)	0.642	0.623	0.686
DT	0.663(0.622 ~ 0.704)	0.671	0.704	0.593
RF	0.714(0.675 ~ 0.753)	0.675	0.690	0.639
xGBoost	0.666(0.625 ~ 0.707)	0.600	0.550	0.711
NB	0.724(0.686 ~ 0.762)	0.634	0.585	0.750
LR	0.665(0.623 ~ 0.707)	0.656	0.690	0.572

Supplementary table 9. Statistical measures of performance of the models in the test cohort; top 15 variables.

Model	AUC (CI 95%)	Accuracy	Sensitivity	Specificity
SVM	0.685(0.645 ~ 0.725)	0.607	0.548	0.745
DT	0.663(0.622 ~ 0.704)	0.671	0.704	0.593
RF	0.728(0.689 ~ 0.766)	0.694	0.713	0.648
xGBoost	0.695(0.656 ~ 0.735)	0.690	0.739	0.576
NB	0.729(0.692 ~ 0.767)	0.658	0.625	0.737
LR	0.659(0.617 ~ 0.702)	0.689	0.773	0.491

Supplementary table 10. Statistical measures of performance of the models in the test cohort; top 20 variables.

Model	AUC (CI 95%)	Accuracy	Sensitivity	Specificity
SVM	0.687(0.647 ~ 0.727)	0.588	0.498	0.800
DT	0.663(0.622~ 0.704)	0.671	0.704	0.593
RF	0.733(0.695 ~ 0.770)	0.681	0.673	0.699
xGBoost	0.689(0.650 ~ 0.728)	0.587	0.505	0.779
NB	0.739(0.702 ~ 0.776)	0.661	0.610	0.754
LR	0.677(0.636 ~ 0.717)	0.661	0.701	0.567

Supplementary table 11. Statistical measures of performance of the models in the test cohort; top 25 variables.

Model	AUC (CI 95%)	Accuracy	Sensitivity	Specificity
SVM	0.667(0.627 ~ 0.707)	0.602	0.539	0.750
DT	0.663(0.622 ~ 0.704)	0.671	0.704	0.593
RF	0.724(0.685 ~ 0.762)	0.659	0.634	0.720
xGBoost	0.698(0.659 ~ 0.737)	0.574	0.463	0.834
NB	0.739(0.702 ~ 0.776)	0.709	0.742	0.631
LR	0.687(0.648 ~ 0.727)	0.663	0.688	0.605

Supplementary table 12. Statistical measures of performance of the models in the test cohort; models for all variables.

Model	AUC (CI 95%)	Accuracy	Sensitivity	Specificity
SVM	0.686(0.647 ~ 0.726)	0.643	0.615	0.707
DT	0.663(0.622 ~ 0.704)	0.671	0.704	0.593
RF	0.727(0.690 ~ 0.764)	0.690	0.712	0.648
xGBoost	0.711(0.672 ~ 0.750)	0.673	0.670	0.682
NB	0.737(0.700 ~ 0.774)	0.654	0.606	0.766
LR	0.694(0.656 ~ 0.733)	0.601	0.529	0.771

Supplementary table 13. Statistical measures of performance of the models in the test cohort; top 5 variables.

Model	AUC (CI 95%)	Accuracy	Sensitivity	Specificity
SVM	0.663(0.629~ 0.697)	0.669	0.697	0.587
DT	0.637(0.602 ~ 0.672)	0.670	0.716	0.538
RF	0.711(0.678 ~ 0.744)	0.674	0.690	0.627
xGBoost	0.692(0.658 ~ 0.727)	0.654	0.650	0.664
NB	0.699(0.666 ~ 0.731)	0.650	0.650	0.652
LR	0.618(0.582 ~ 0.654)	0.606	0.616	0.578

Supplementary table 14. Statistical measures of performance of the models in the test cohort; top 10 variables.

Model	AUC (CI 95%)	Accuracy	Sensitivity	Specificity
SVM	0.701(0.667 ~ 0.735)	0.720	0.777	0.556
DT	0.637(0.602 ~ 0.672)	0.670	0.716	0.538
RF	0.754(0.722 ~ 0.786)	0.670	0.640	0.750
xGBoost	0.710(0.676 ~ 0.744)	0.646	0.613	0.741
NB	0.721(0.689 ~ 0.753)	0.655	0.627	0.735
LR	0.618(0.582 ~ 0.654)	0.640	0.616	0.640

Supplementary table 15. Statistical measures of performance of the models in the test cohort; top 15 variables.

Model	AUC (CI 95%)	Accuracy	Sensitivity	Specificity
SVM	0.710(0.677 ~ 0.744)	0.684	0.705	0.640
DT	0.637(0.602 ~ 0.672)	0.670	0.716	0.538
RF	0.778(0.747 ~ 0.808)	0.759	0.797	0.649
xGBoost	0.733(0.700 ~ 0.766)	0.709	0.729	0.649
NB	0.705(0.671 ~ 0.738)	0.629	0.585	0.756
LR	0.676(0.642 ~ 0.710)	0.629	0.617	0.664

Supplementary table 16. Statistical measures of performance of the models in the validation cohort; top 25 variables.

Model	AUC (CI 95%)	Accuracy	Sensitivity	Specificity
SVM	0.723(0.690 ~ 0.755)	0.659	0.635	0.726
DT	0.637(0.602 ~ 0.672)	0.670	0.716	0.538
RF	0.777(0.746 ~ 0.807)	0.739	0.756	0.689
xGBoost	0.736(0.703 ~ 0.769)	0.751	0.815	0.566
NB	0.722(0.689 ~ 0.754)	0.628	0.572	0.790
LR	0.686(0.653 ~ 0.720)	0.673	0.693	0.615

Supplementary table 17. Statistical measures of performance of the models in the validation cohort; models for all variables.

Model	AUC (CI 95%)	Accuracy	Sensitivity	Specificity
SVM	0.690(0.658 ~ 0.723)	0.554	0.456	0.833
DT	0.637(0.602 ~ 0.672)	0.670	0.716	0.539
RF	0.770(0.740 ~ 0.801)	0.750	0.794	0.630
xGBoost	0.735(0.702 ~ 0.767)	0.668	0.654	0.707
NB	0.715(0.682 ~ 0.747)	0.677	0.679	0.670
LR	0.686(0.653 ~ 0.720)	0.641	0.645	0.630
